# Review on blueberry drought tolerance from the perspective of cultivar improvement

**DOI:** 10.3389/fpls.2024.1352768

**Published:** 2024-05-14

**Authors:** Sushan Ru, Alvaro Sanz-Saez, Courtney P. Leisner, Tanzeel Rehman, Savannah Busby

**Affiliations:** ^1^ Department of Horticulture, Auburn University, Auburn, AL, United States; ^2^ Department of Crop, Soil and Environmental Sciences, Auburn University, Auburn, AL, United States; ^3^ School of Plant and Environmental Sciences, Virginia Polytechnic Institute and State University, Blacksburg, VA, United States; ^4^ Department of Biosystems Engineering, Auburn University, Auburn, AL, United States

**Keywords:** *Vaccinium* spp., breeding, cultivar evaluation, chlorophyll fluorescence, high-throughput phenotyping

## Abstract

Blueberry (*Vaccinium* spp.) is an increasingly popular fruit around the world for their attractive taste, appearance, and most importantly their many health benefits. Global blueberry production was valued at $2.31 billion with the United States alone producing $1.02 billion of cultivated blueberries in 2021. The sustainability of blueberry production is increasingly threatened by more frequent and extreme drought events caused by climate change. Blueberry is especially prone to adverse effects from drought events due to their superficial root system and lack of root hairs, which limit blueberry’s ability to intake water and nutrients from the soil especially under drought stress conditions. The goal of this paper is to review previous studies on blueberry drought tolerance focusing on physiological, biochemical, and molecular drought tolerance mechanisms, as well as genetic variability present in cultivated blueberries. We also discuss limitations of previous studies and potential directions for future efforts to develop drought-tolerant blueberry cultivars. Our review showed that the following areas are lacking in blueberry drought tolerance research: studies of root and fruit traits related to drought tolerance, large-scale cultivar screening, efforts to understand the genetic architecture of drought tolerance, tools for molecular-assisted drought tolerance improvement, and high-throughput phenotyping capability for efficient cultivar screening. Future research should be devoted to following areas: (1) drought tolerance evaluation to include a broader range of traits, such as root architecture and fruit-related performance under drought stress, to establish stronger association between physiological and molecular signals with drought tolerance mechanisms; (2) large-scale drought tolerance screening across diverse blueberry germplasm to uncover various drought tolerance mechanisms and valuable genetic resources; (3) high-throughput phenotyping tools for drought-related traits to enhance the efficiency and affordability of drought phenotyping; (4) identification of genetic architecture of drought tolerance using various mapping technologies and transcriptome analysis; (5) tools for molecular-assisted breeding for drought tolerance, such as marker-assisted selection and genomic selection, and (6) investigation of the interactions between drought and other stresses such as heat to develop stress resilient genotypes.

## 1 Drought stress presents a major threat to global blueberry production

Blueberry (*Vaccinium* spp.) is an increasingly popular fruit around the world for their attractive taste, appearance, and most importantly their many health benefits. Blueberries contain high amounts of fiber, minerals, and organic acids (Silva et al., 2020), which may contribute to reducing inflammation (Silva et al., 2020), preventing cardiovascular disease, maintaining blood sugar levels (Zou et al., 2022), and slowing memory loss and the loss of fine motor skills (Evans and Ballen, 2014). Driven by consumer demand, global blueberry production has more than doubled between 2010 and 2021 from 439,000 metric tons to 1.1 million metric tons (FAOSTAT, accessed on 2023-08-23). Global blueberry production was valued at $2.31 billion (FAOSTAT, accessed on 2023-08-23) with the United States alone producing $1.02 billion and 40,225 hectares (99,400 acres) of cultivated blueberries in 2021 (USDA NASS, accessed on 2022-07-01). Cultivated blueberries, originated from North America, belong to the section Cyanococcus under the genus *Vaccinium*. Most commercial cultivars are descendants of highbush blueberries, which can be classified into northern highbush blueberries (*Vaccinium corymbosum* L., 2*n* = 4*x* = 48) for their high chilling requirements and winter hardiness, and southern highbush blueberries (derived from crossing *Vaccinium corymbosum* L., V. *darrowi* Camp, and other *Vaccinium* species, 2*n* = 4*x* = 48), which are adjusted to low-chilling hours and warmer climates (Hancock et al., 2008). Additionally, rabbiteye blueberry (*Vaccinium virgatum* A., 2*n* = 6*x* = 72) is a native species in the Southeast United States and is mainly grown for retail production for its high yield and tolerance to a wide range of biotic and abiotic stresses (Scherm and Krewer, 2003). Blueberries exhibit primarily outcrossing behavior with different degrees of self-fertility, which is the highest for northern highbush blueberries (NHB), followed by southern highbush blueberries (SHB), and rabbiteye blueberries (RE) (Hancock et al., 2008).

**Figure 1:**
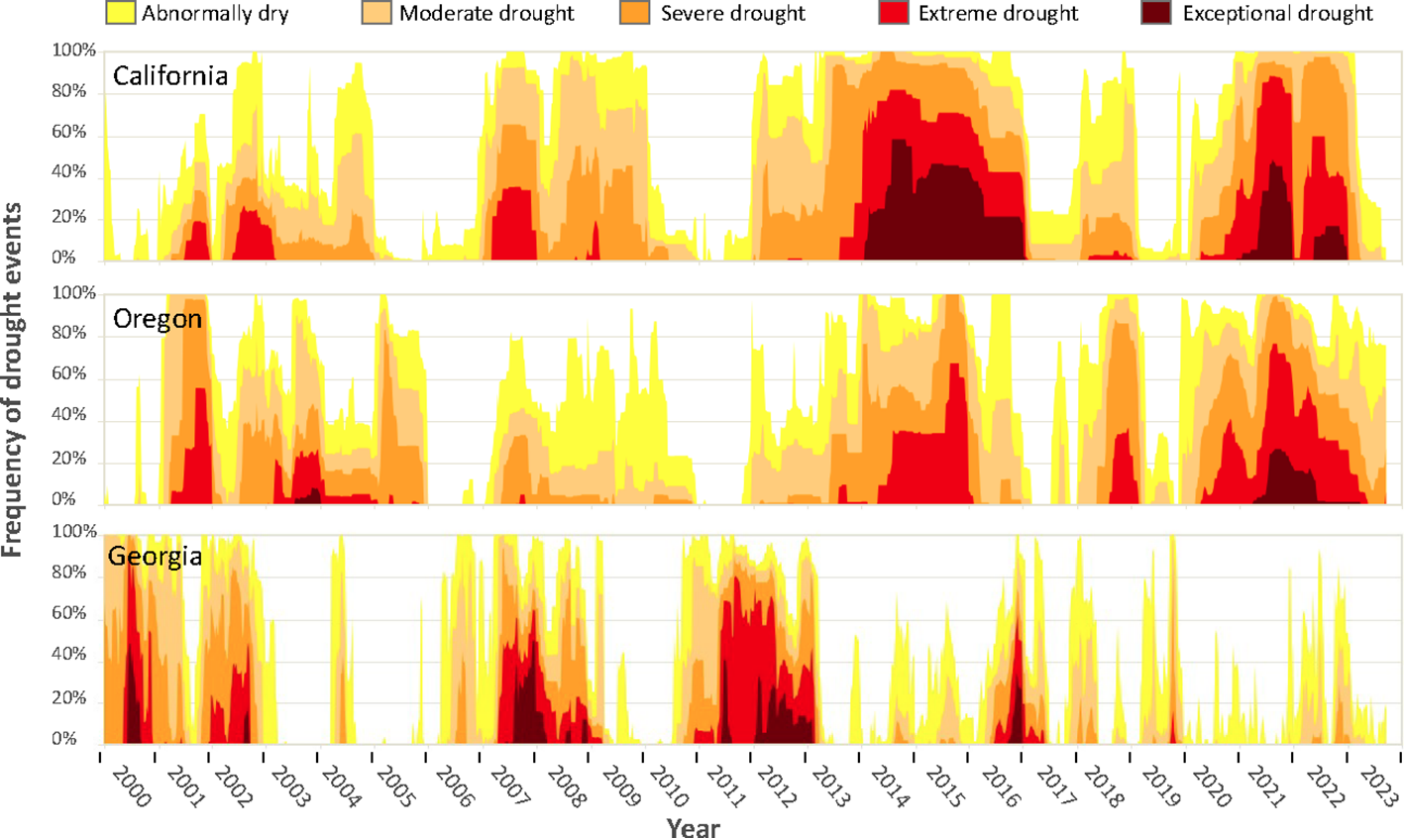
Time series of drought events in California, Oregon, and Georgia from 2000 to 2024. Graphs obtained from U.S. Drought Monitor (https://droughtmonitor.unl.edu/DmData/TimeSeries.aspx, accessed on September 25, 2023). According to U.S. Drought Monitor, drought intensity was classified into five categories from abnormally dry (D0) as the least severe category to exceptional drought (D4) as the most severe category. Classification was based on standard precipitation index (SPI) and standardized precipitation-evapotranspiration index (SPEI). More explanation of drought classification can be found at: https://droughtmonitor.unl.edu/About/AbouttheData/DroughtClassification.aspx. The U.S. Drought Monitor is jointly produced by the National Drought Mitigation Center at the University of Nebraska-Lincoln, the United States Department of Agriculture, and the National Oceanic and Atmospheric Administration. Map courtesy of NDMC.

As the blueberry industry continues to expand, the sustainability of blueberry production is increasingly threatened by more frequent and extreme drought events caused by climate change (Li et al., 2009). Agricultural drought occurs when levels of soil moisture decrease, which can lead to soil-water stress for crops and put a major constraint on agricultural production (Lu et al., 2020). Loss of soil-water availability can quickly lead to crop failure, lower yields, and ultimately threaten food security (Lu et al., 2020). Rising global temperatures disrupt the hydrological cycle, and therefore cause more water-related stress for crops such as drought and flood (Mukherjee et al., 2018). As a result, drought disaster-affected areas are predicted to increase from 15.4 to 44% by 2100 on a global scale, together with a doubled drought risk index (Li et al., 2009). Increasing drought events are also impacting major blueberry production regions. For example, in the United States, three of the top five blueberry producers are all subject to frequent drought events (California (No. 4), Oregon (No. 2), and Georgia (No. 3) (**Figure 1**) (USDA NASS, accessed on 2022-07-01; U.S. Drought Monitor, accessed on 2023-09-25).

Blueberry is especially prone to adverse effects from drought events (Erb, 1993; Améglio et al., 2000; Molnar et al., 2022) (**Figure 1**). Limited drought tolerance of blueberries is a byproduct of their superficial root system, with most blueberries growing roots less than 40 cm deep (Molnar et al., 2022) (**Figure 1**). Additionally, blueberries lack root hairs, which reduces their absorptive root surface by 90% compared to root systems with root hairs (Eck, 1966; McElrone et al., 2013). As a result, blueberries have a limited ability to intake water and nutrients from the soil especially under drought stress (Erb, 1993). While cultivated blueberries such as NHB (*V. corymbosum*) and SHB (*V. corymbosum* interspecific hybrids) are most susceptible to drought stress, the genetic variability present in other *Vaccinium* species makes it promising to improve cultivated blueberries for better drought tolerance. For example, the wild blueberry species such as lowbush blueberries (*V*. *angustifolium* Ait.), *V. darrowii*, and *V. elliottii* are known to be drought resistant (Erb, 1993). The RE blueberry (*V. virgatum*) is considered more drought tolerant than highbush blueberries (Erb, 1993). The goal of this paper is to review previous studies (**Supplementary Tables S1**-**S3**) on drought tolerance in cultivated blueberries focusing on the following areas:

1. Impact of drought on blueberries’ physiological and biochemical performance,
2. Impact of drought on blueberry growth and fruit production,
3. Blueberries’ molecular responses to drought stress
4. Variation in drought tolerance in cultivated blueberry cultivars, and
5. Limitations of previous studies and directions of future research to develop drought tolerant cultivars.

## 2 Impact of drought on the physiological and biochemical performance of blueberries

**Figure 2:**
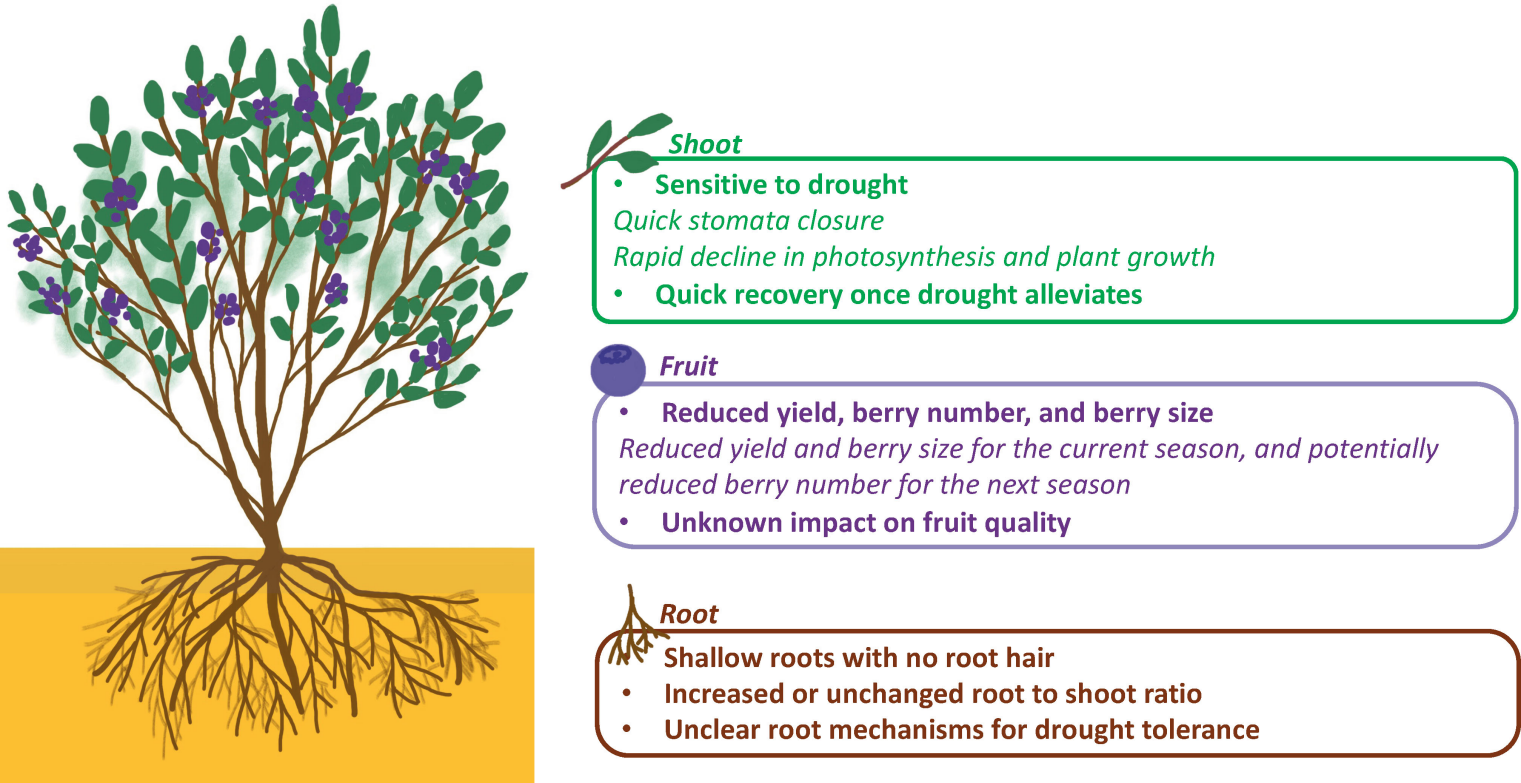
Characteristics of blueberry’s shoots, fruits, and roots in response to drought. The summarized characteristics of each plant part are highlighted in bold, while examples of drought responses are denoted in italics.

**Figure 3:**
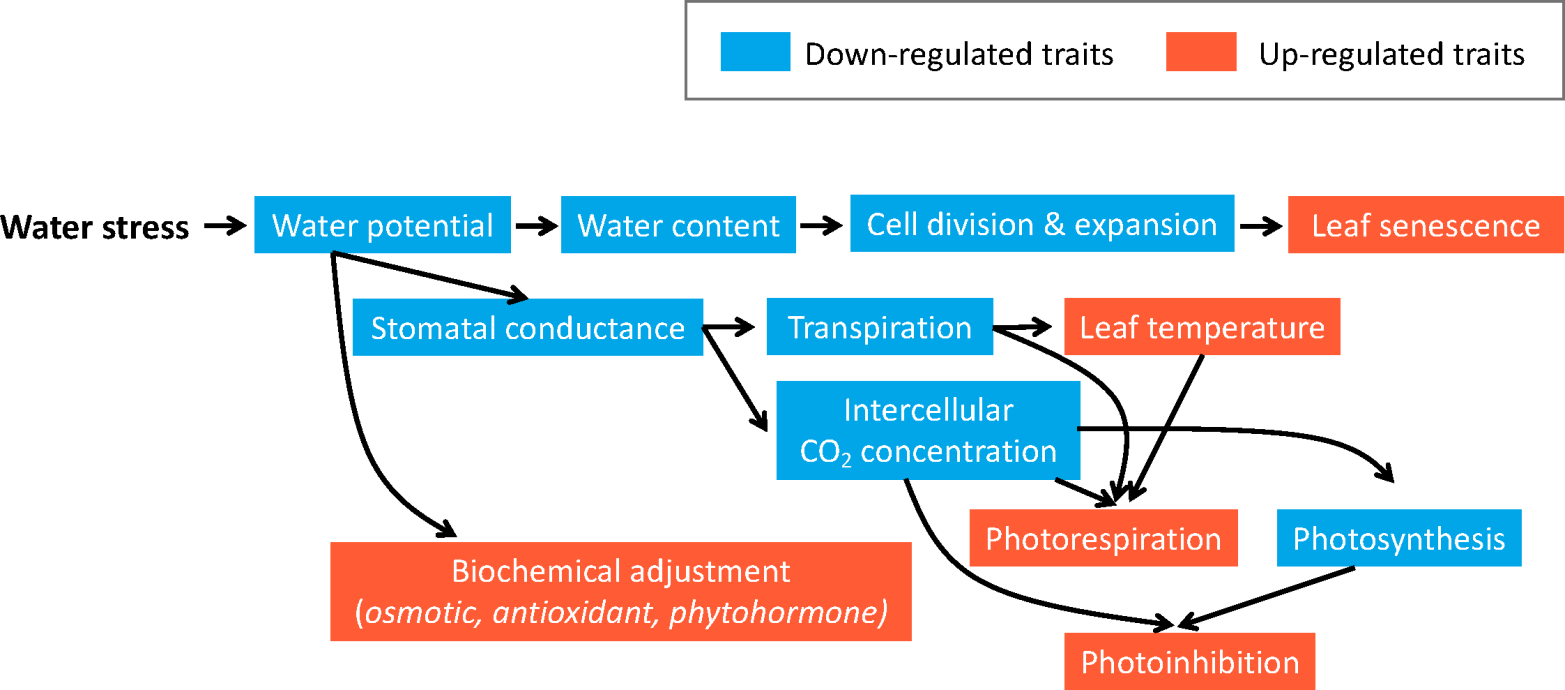
Blueberries’ physiological response to drought in the shoots. The blue color signifies down-regulated traits, while orange represents up-regulates traits. When blueberry plants experience drought stress, their water potential decreases, leading to reduced water content in cells. Consequently, slowdown in cell division and expansion negatively impacts leaf growth and may induce leaf senescence. Furthermore, low water potential causes stomatal closure to decrease transpiration and intercellular CO_2_ concentration, thereby limiting photosynthesis. Decreased transpiration and CO_2_ concentration, along with increased leaf temperature, can induce photorespiration. In the meantime, decreased photosynthesis activity and CO_2_ concentration may result in photoinhibition. Furthermore, water stress can also cause biochemical adjustment, including osmotic, antioxidant, and phytohormone adjustment to protect plants from ROS and other damages caused by drought stress.

Two major mechanisms have been observed in blueberries to cope with drought stress. One is through stomatal closure to reduce water loss, the other is through osmotic adjustment to maintain water absorption and cell turgor under water deficiency (Erb, 1993) (**Figure 2**). Blueberries are considered isohydric plants, which can maintain a constant leaf water potential under drought stress by adjusting stomatal opening/closure to control water loss. As a result, stomatal closure under drought can protect blueberries from severe damages such as xylem embolism, but this can also limit the intake of CO_2_ and therefore reduce photosynthesis rate, negatively affecting plant growth and production (Sade et al., 2012). Another major mechanism of plants to alleviate drought stress is to increase water uptake with prolific root systems (Erb, 1993; Farooq et al., 2012); however, this mechanism is poorly understood in blueberries due to the difficulties of phenotyping roots especially under field conditions (**Figure 3**). Here we mainly focus on physiological, biochemical, and post-harvest changes in the blueberry canopy in response to drought stress. Commonly studied traits related to drought response and their references were listed in **Table 1**.

**Table 1:** Commonly studied traits related to drought response in cultivated blueberries.

Category	Trait	References
Physiology	Leaf/stem water potential	Davies and Johnson, 1982; Améglio et al., 2000
	Relative water content (RWC)	Davies and Johnson, 1982; Chen et al., 2017; Balboa et al., 2020
	Stomatal conductance	Améglio et al., 2000; Perrier et al, 2000; Mingeau et al., 2001
	Transpiration	Davies and Johnson, 1982; Améglio et al., 2000; Perrier et al, 2000; Mingeau et al., 2001
	Total chlorophyll content	Chen et al., 2017; Mazurek et al., 2021
	Carotenoids	Molnar et al., 2022
	Net photosynthetic rate	Chen et al., 2017
	Relative electrolyte conductivity (REC)	Chen et al., 2017
	Carbon isotopic discrimination (Δ^13^C)	Balboa et al., 2020
	Basic chlorophyll fluorescence yield (F_0_) Maximum fluorescence (Fm) Variable fluorescence (Fv) Water uptake (Fv/F_0_)	Estrada et al., 2015; Balboa et al., 2020; Mazurek et al., 2021
	Maximum photochemical quantum yield of PSII (Fv/Fm) Maximum rate of electron transport (ETR_max_) Non-photochemical quenching (NPQ)	Estrada et al., 2015
Biochemical	Superoxide dismutase (SOD) activities	Chen et al., 2017
	Peroxidase (POD) activities	Chen et al., 2017
	Total soluble sugar contents of blueberry leaves	Chen et al., 2017
	Proline content	Balboa et al., 2020
	Abscisic acid (ABA) content	Chen et al., 2017
	Indole-3-acetic acid (IAA) content	Chen et al., 2017
	Gibberellic acid (GA_3_) content	Chen et al., 2017
	Polyamine (PA) content	Chen et al., 2017
	Malondialdehyde (MDA) content	Chen et al., 2017
	Dehydrin proteins content	Panta et al., 2001
Growth & fruit production	Stem diameter	Perrier et al., 2000; Mingeau et al., 2001
	Shoot elongation	Mingeau et al., 2001; Mazurek et al., 2021
	Canopy and root dry weight	Davies and Johnson, 1982
	Specific leaf weight	Chen et al., 2017
	Specific leaf area	*Balboa et al., 2020*
	Yield in the current year	Perrier et al., 2000; Mingeau et al., 2001
	Average berry weight in the current year	Perrier et al., 2000; Mingeau et al., 2001
	Berry number	Perrier et al., 2000; Mingeau et al., 2001
	Berry number next year	Perrier et al., 2000; Mingeau et al., 2001
	Average berry weight in the next year	Perrier et al., 2000; Mingeau et al., 2001
	Yield in the next year	Perrier et al., 2000; Mingeau et al., 2001

### 2.1 Stomatal closure and subsequent impacts on photosynthesis

Among all the physiological processes that are essential to plant growth and production, photosynthesis is one of the most affected traits under drought (Cameron et al., 1989; Farquhar et al., 1989; Condon et al., 2004; Rho et al., 2012) (**Figure 2**). Photosynthesis is composed of several interconnected physical and biochemical processes that result in CO_2_ fixation in mesophyll cells followed by the accumulation of sugars, cellular components, and biomass. The light and CO_2_ assimilation reactions are impacted by drought via different mechanisms including 1) biophysical limitations caused by stomatal closure (Cornic, 2000) and limited CO_2_ diffusion through the mesophyll (Sharkey et al., 2007), and 2) biochemical limitations such as reductions in the electron transport chain in the thylakoid (Estrada et al., 2015; Pilon et al., 2018) or decreased activity of the enzyme ribulose-1,5-bisphosphate carboxylase/oxygenase (Rubisco) (Flexas and Medrano, 2002; Lawlor and Cornic, 2002; Flexas et al., 2006; Morales et al., 2020).

Blueberries can show physiological changes as soon as three to four days after drought stress occurs (Améglio et al., 2000). First, leaf and stem water potential will quickly drop in the presence of water deficiency (Davies and Johnson, 1982; Améglio et al., 2000). Decrease in water potential will then trigger rapid closure of stomata to reduce transpiration and therefore limit water loss (Améglio et al., 2000; Perrier et al., 2000; Mingeau et al., 2001). Reduced stomatal conductance can help plants to maintain the minimum water potential at a stable level and therefore avoid severe damage such as xylem embolism (Améglio et al., 2000). As a result of stomatal closure, the photosynthetic rate tends to decline due to decreased intercellular CO_2_ concentration and other changes (e.g., reduced chlorophyll content) caused by drought (Sade et al., 2012). In blueberry, this quick stomatal closure is mediated by increases in the abscisic acid sent from the roots to the shoots (Farooq et al., 2012; Chen et al., 2017). In an experiment performed in SHB blueberries, Chen et al. (2017) observed that as drought progressed during a 16-days period, photosynthesis decreased due to stomatal closure. Decrease in photosynthesis and stomatal conductance in blueberries under drought stress has also been reported in multiple studies based on measuring leaf photosynthesis using a leaf infrared gas analyzer (LI-6400/XT Portable Photosynthesis System, Lincoln, NE, United States: LI-COR Biosciences; Cameron et al., 1989; Améglio et al., 2000; Chen et al., 2017).

Carbon isotope discrimination (Δ^13^C) is another effective way to reflect how drought reduces stomatal conductance and photosynthesis, in addition to directly measuring stomatal conductance and photosynthetic capacity. Three forms of carbon isotopes are present in the atmosphere with ^12^C and ^13^C being the stable isotopes and ^14^C a radioactive isotope (Graven et al., 2020). For the stable isotopes, the light isotope ^12^C is more abundant than the heavy stable isotope, ^13^C. In plants, ^12^C is preferably fixed by Rubisco during photosynthesis because of its abundance and smaller size in comparison with ^13^C (Farquhar et al., 1989). Under drought-triggered stomatal closure, there is less opportunity for ^13^C to diffuse out of the stomatal cavity leading to greater assimilation of this isotope, and higher concentrations of ^13^C in plant dry matter (Farquhar et al., 1982). Δ^13^C is defined as the deviation of isotope effects (*α*) from the unity, which equals *α* −1 = *R_r_*/*R_p_*−1, where *R_r_* is the ^13^C/^12^C molar ratio of reactant and *Rp* is the molar ratio of products (Farquhar et al., 1989). In general, when plants fix more ^13^C, there is less C^13^ discrimination and therefore a lower Δ^13^C. Δ^13^C has been used widely to study the effects of drought on stomatal closure for its effectiveness in reflecting stomatal behavior during the whole growing season, which is powerful especially when gas exchange measurements are not feasible (Farquhar et al., 1989; Condon et al., 2004). In blueberry, Balboa et al. (2020) showed that drought decreased the Δ^13^C values in all studied cultivars. These authors observed that some cultivars such as Brigitta and Biloxi had lower Δ^13^C than Sharpblue. As low Δ^13^C has been related with high water use efficiency and drought tolerance, the Δ^13^C discrimination technique could be used to screen for these kinds of traits in the future (Condon et al., 2004; Zhang et al., 2022; *see drought tolerance section*).

### 2.2 Photorespiration and photoinhibition

Following drought-induced stomatal closure, photorespiration can be triggered due to the combination of low intercellular CO_2_, low transpiration rate, and high leaf temperature (**Figure 2**). During photorespiration, the enzyme Rubisco will oxygenate ribulose-1,5-bisphosphate (RuBP) to produce glycerate-3-phosphate, and 2-phosphoglycolate. As a result, photorespiration decreases the efficiency of photosynthesis and produces reactive oxygen species (ROS) such as such as O_2_^-^, O_2_^1^, H_2_O_2_, RO and OH^-^ that can damage membranes and enzymes (Chastain et al., 2014; Gahir et al., 2021). In addition to photorespiration, reduced CO_2_ fixation under drought also leads to an accumulation of photons as the plant is still harvesting light regardless of lower photosynthetic rate to utilize the light energy. Excessive photons accumulated in drought-stressed plants will give rise to photoinhibition (**Figure 2**), which, according to Long et al. (1994), is “the light independent and slowly reversible retardation of photosynthesis, independent of any developmental change”. Photoinhibition is a mechanism to protect plants from light damage to photosystem II (PSII) (Long et al., 1994). In addition, photoinhibition can also decrease photosynthesis efficiency and produces ROS, similar to photorespiration (Krause, 1988). ROS can damage membrane structures and proteins in the thylakoids and therefore reduce the light reactions of photosynthesis (Maxwell and Johnson, 2000; Chastain et al., 2014; Pilon et al., 2018; Liang et al., 2019).

### 2.3 Changes in chlorophyll fluorescence

The efficiency of light reactions can be measured with chlorophyll fluorescence analysis. Light energy absorbed by chlorophyll can be used to drive photosynthesis reactions, lost as heat, or re-emitted as light (Maxwell and Johnson, 2000). Chlorophyll fluorescence measures how much light energy is re-emitted as light by chlorophyll, which can provide insight into the efficiency of photochemistry (Maxwell and Johnson, 2000). In blueberries, as water stress advances and the leaf water potential decreases, the light reactions also get damaged and fluoresce parameters such as quantum yield of PS II (ϕPSII) and photosynthetic electron transport rate (ETR) are significantly reduced (Estrada et al., 2015; Balboa et al., 2020). Estrada et al. (2015) and Balboa et al. (2020) observed differences in fluorescence parameters among various cultivars under drought, which suggests the potential to select cultivars based on drought-resistant photochemistry (*see drought tolerance section*). In addition to blueberries, drought impacts on fluorescence parameters were also observed in crops such as cotton (Chastain et al., 2014), peanut (Pilon et al., 2018), lettuce (Shin et al., 2021), and tomato (Liang et al., 2020). In lettuce seedlings, drought stress impacted chlorophyll fluorescence parameters especially under severe drought (Shin et al., 2021). In tomatoes, chlorophyll fluorescence parameters like the maximum quantum yield (F_V_/F_M_) and ϕPSII declined as drought stress increased, indicating closure of the PSII reaction center and reduced electron transfer (Liang et al., 2020). Although chlorophyll fluorescence provides an estimate of photosynthetic conditions, it is best combined with other techniques like gas exchange measurements because chlorophyll fluorescence does not directly measure carbon fixation (Maxwell and Johnson, 2000).

In many cases the decrease in fluoresce parameters (Estrada et al., 2015; Balboa et al., 2020) is also related to the decrease in chlorophyll and carotenoid content during drought (Chen et al., 2017; Mazurek et al., 2021; Molnar et al., 2022). Additionally, membrane damage as result of ROS accumulation can also be monitored through malondialdehyde (MDA) content, which is a product of membrane lipid peroxidation because of membrane damage (Ma et al., 2015). In a study of highbush blueberry seedlings, Chen et al. (2017) observed that drought stress increased the levels of MDA, which is likely due to membrane damage and lipid peroxidation. Membrane damage under drought can also be studied by measuring the relative electrolyte conductivity (REC) which signals electrolyte leakage due to pores in the membranes (Demidchik et al., 2014). As drought advances in blueberries, an increase in relative electrolyte conductivity can be observed, together with increased MDA and decreased ϕPSII and chlorophyll content (Chen et al., 2017).

### 2.4 Biochemical responses to drought

Plants have evolved a complex antioxidant system to protect cells from ROS damages by producing enzymatic and non-enzymatic antioxidants to act as ROS scavengers (Farooq et al, 2012). Examples of enzymatic antioxidants include superoxide dismutase (SOD), catalase (CAT), peroxidase (POD), and non-enzymatic antioxidants include polyamine (PA), salicylates, and compatible solutes including proline, and glycinebetaine (GB) (Farooq et al, 2012; Chen et al., 2017). An increase in the activities of SOD and POD was reported in blueberries under drought stress (Panta et al., 2001; Wu et al., 2016; Chen et al., 2017). In the case of SOD there was significant cultivar response in this parameter to drought, which could therefore indicate that cultivars with higher SOD could provide drought tolerance as they have more ROS scavenger capacity than other (Wu et al., 2016; *see drought tolerance section*).

When drought continues for a long period of time, the leaf water potential needs to remain low so that water can be drawn into the leaf from the root and maintain turgor. To do this, plants concentrate osmolytes that can be organic (soluble sugars, organic acids, and amino acids) and inorganic (ions such as Na^+^, K^+^, Ca2^+^, and Cl^–^) (Farooq et al., 2012). It has been demonstrated in blueberries that as drought progresses, leaves accumulate active osmolytes such as proline (Balboa et al., 2020) and soluble sugars (Chen et al., 2017) to decrease the osmotic potential and thus the leaf water potential.

## 3 Impact of drought on blueberry growth and fruit production

As a result of impacted photosynthesis rate and other physiological, biochemical, and molecular changes caused by water deficiency, drought will show both short-term and long-term effects on plant growth parameters and fruit production (Perrier et al., 2000; Mingeau et al., 2001). For example, specific leaf area, which is the ratio of leaf area to leaf dry mass, tends to decrease during drought (Balboa et al., 2020). Specifically, individual leaf area tends to decrease, and the shape of leaves tend to become narrower and thicker in response to drought with the objective of reducing transpiration (Cameron et al., 1989; Balboa et al., 2020). In addition, shoot elongation and stem diameter, are negatively impacted by drought due to reduction in turgor and photosynthesis (Perrier et al., 2000; Mingeau et al., 2001). In many crops, drought tends to decrease shoot growth and promote root growth to reduce transpiration and maximize water uptake (Farooq et al., 2012). In blueberries, higher root to shoot ratio was observed in NHB cultivars Jersey and Bluecrop in response to drought (Cameron et al., 1989) whereas no change was observed in the RE cultivar Bluegem (Davies and Johnson, 1982). More research is needed however, to understand root response to drought among various ecotypes and cultivars grown under the same field conditions. Under severe drought stress, irreversible damages such as premature leaf senescence and xylem embolism could occur and eventually cause plant death (Farooq et al., 2012; Salehi-Lisar and Bakhshayeshan-Agdam, 2016).

**Figure 4:**
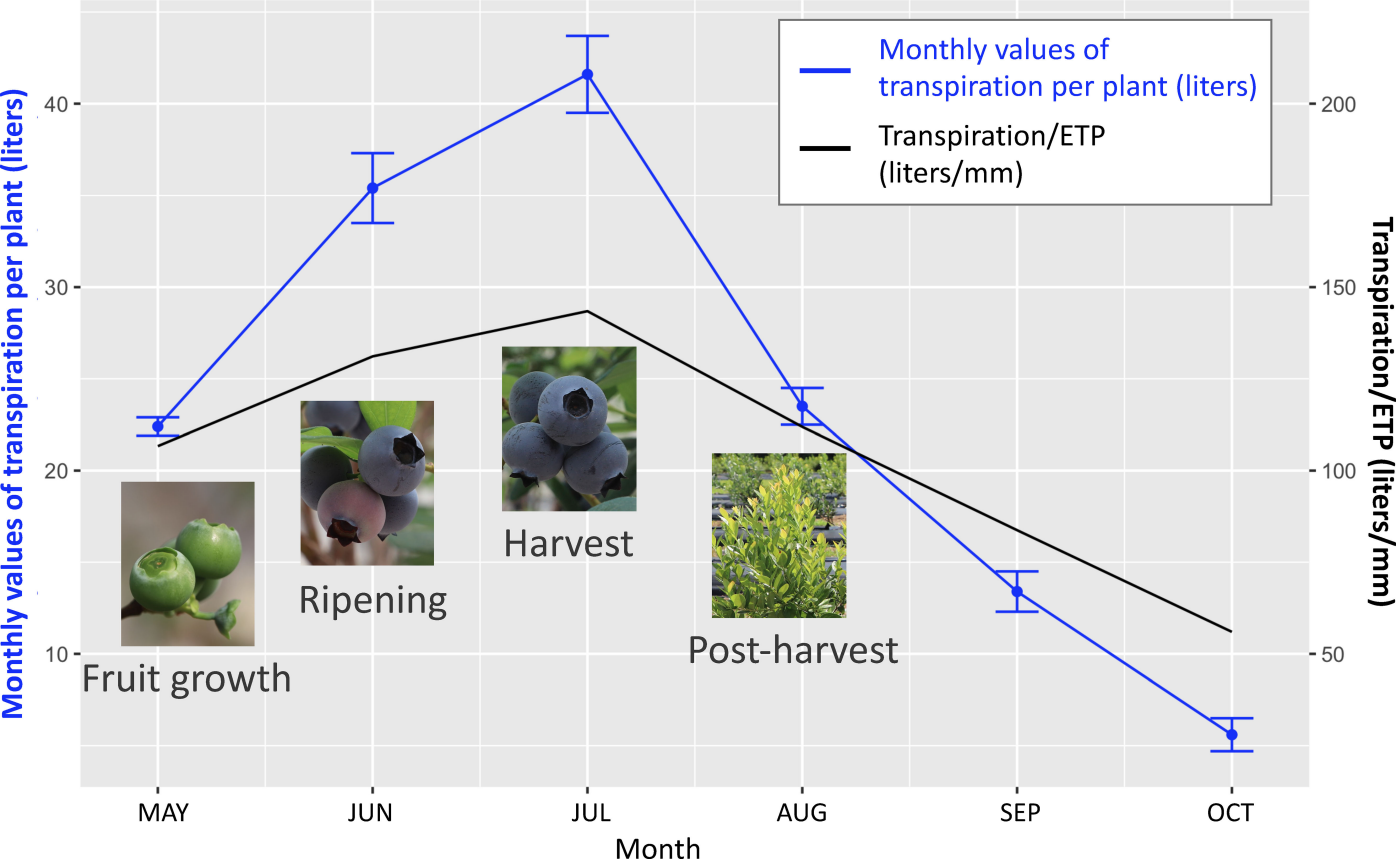
Water need varies across blueberry growth stages. The left Y-axis stands for monthly values of transpiration per plant (liters), data of which is shown with the blue line with error bars. The right Y-axis stands for monthly values of transpiration per plant divided by the estimated net evapotranspiration from meteorological data (ETP) (liters/mm). Ripening and harvest are the most water-demanding stages during a growing season. Data based on Mingeau et al. (2001).

**Figure 5:**
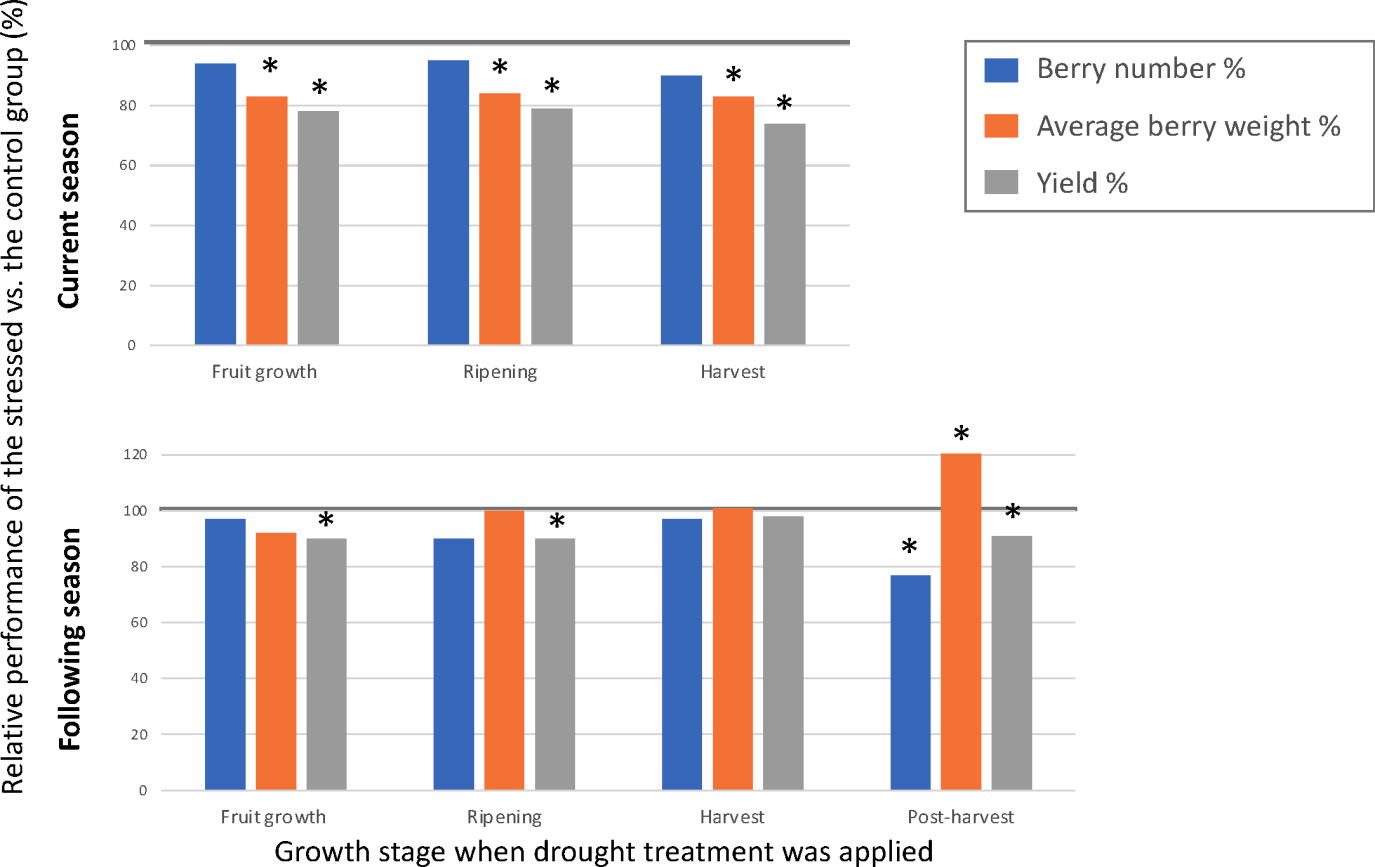
Effect of growth stage on drought sensitivity. Data based on Mingeau et al. (2001). Effect of timing of drought for the current season (top chart) and following season (bottom chart). Y-axis stands for the percentage of berry number, average berry weight (g), and yield (g) under moderate drought treatment versus well-watered control group. X-axis stands for the growth stage when drought treatment was applied. Bars with * were significantly different from the control group (*p* value < 0.05).

Existing knowledge on drought effects on fruit-related traits is scarce compared to the response of physiological traits to drought, as the majority of the physiological research has been performed on juvenile plants that did not produce berries. Perrier et al. (2000) and Mingeau et al. (2001) are the only studies which evaluated drought effect on yield, berry number, and berry size. Authors of these two studies applied drought treatments during various stages of plant growth of the blueberry cultivar Bluecrop and evaluated its effects on fruit production in the same and the subsequent years. Both studies found that drought stress during fruit growth, ripening, or harvest can significantly reduce yield and average berry weight in the same year. The amount of water used by a well-watered plant was the highest during ripening and harvest during a year, whereas the lowest during post-harvest (**Figure 4**). Drought occurring during the fruit growth stage had the largest impact on yield (20-49% less) and average berry weight (17-39% less) in the same season, whereas drought during post-harvest growth had the largest impact on berry number (23-43% less), average berry weight (21-59% higher), and yield (reduced for up to 27%) in the following season (**Figure 5**). Due to drought’s impact on berry number in the following season, it was suggested that moderate post-harvest water stress can be used as a management practice to control fruit load and increase berry size to save the cost of pruning (Perrier et al., 2000; Mingeau et al., 2001).

Besides yield and average berry weight, fruit quality traits such as sugar, acidity content, and fruit texture are even more important factors in determining the profitability of the fresh blueberry market. Drought was found to impact fruit quality traits such as size, firmness, sugars, acids, and sugar/acid ratio in many deciduous fruit crops (e.g., apple, pear, and peach) (Lopez et al., 2011). Soluble solids concentration (SSC) tends to increase under drought in crops such as apple, pear, peach, and plum (Lopez et al., 2011). On the other hand, drought effects on fruit acidity tended to vary across crops, cultivars, or even growing seasons of the same plant (Lopez et al., 2011). In peach, plants under drought stress were reported to have smaller fruit, increased fruit firmness, higher sweetness index and malic acid concentration, but lower citric and uinic acid concentration (Rahmati et al., 2015). In apple, water deficits during fruit expansion phases were found to be most impactful on the apple cultivar Honeycrisp, compared to early season treatment (Reid and Kalcsits, 2020). Honeycrisp apples under drought stress tended to have smaller, firmer, and sweeter fruit in some of the growing seasons (Reid and Kalcsits, 2020). However, information on how drought affects fruit quality has not been reported in blueberries. Additionally, drought has been reported to negatively affect plant phenology in many crops, such as wheat and barley, by shortening the growth cycle (McMaster and Wilhelm, 2003). However, few studies have compared phenological stages between drought-stressed and well-watered treatments in blueberries.

## 4 Blueberries’ molecular responses to drought

Plants’ physiological, biochemical, and postharvest responses to drought are ultimately regulated at the genetic and molecular level. This occurs through the presence/absence of specific genes, regulation of gene expression (transcriptionally, post-transcriptionally) and ultimately protein synthesis. Transcription factors (TFs) are regulatory elements (proteins or DNA-binding factors) that can enhance or suppress expression of genes due to developmental cues or environmental signals. Recent work has demonstrated the role of MYB TFs in response to stress from drought. For example, Zhang et al. (2020) found that the blueberry *VcMYB4a* gene is downregulated by drought, salt, and cold stresses and that overexpression of the *VcMYB4a* gene in blueberry callus increased sensitivity to drought and other stresses (Zhang et al., 2020). Additionally, a recent study was published analyzing the expression of MYB TFs in response to drought in the NHB blueberry cultivar Bluecrop (Wang et al., 2021). This study identified 229 non-redundant MYB sequences (VcMYB) in the blueberry genome. Following identification, differential expression analysis of the *VcMYBs* under drought stress was performed. From this, a total of 102 differentially expressed genes (DEGs) were identified in leaves and roots. Analysis of the interacting partners of key VcMYB proteins found *VcMYB* genes are likely involved in the ROS signaling pathway and leaf morphology and structure under drought stress (Wang et al., 2021). In addition, Wang et al. (2022) exposed the Bluecrop cultivar to moderate and severe drought stress then performed RNA-sequencing, differential gene expression and co-expression analysis to determine genes involved in blueberry responses to drought. This study reinforced that TFs played an important role in blueberry response to drought and highlighted that roots and leaves of blueberries exposed to drought stress had unique gene expression patterns (Wang et al., 2022). The TFs most likely involved in drought stress in leaves and roots in Bluecrop were *VcABR1*, *VcABF2*, *VcMYB108*, and *VcMYB93* (Wang et al., 2022). This further underscores the importance of the MYB family of transcription factors in addition to the AP2/ERF transcription factor family in blueberry responses to drought (Wang et al., 2022).

The NAC (NAM, ATAF1, ATAF2, CUC2) TF family has also been shown to be involved in drought response in blueberry (Liang et al., 2019). The NAC TF family is known to have an extensive role in both plant development, hormone signaling pathways, and stress response (Liang et al., 2019). A total of 158 NACs were identified in the blueberry genome and 62 NACs were differentially expressed in root and leaf tissues in response to drought in blueberry. Of those 62 DEGs, 33 were identified as being differentially expressed in the roots and 51 in the leaves in response to drought, with 22 being co-expressed in both roots and leaves in response to drought (Liang et al., 2019). In addition, an expression correlation network for the 62 identified differentially expressed NAC genes found that several of the NAC TFs were associated with one another, suggesting the possibility that these NAC genes could potentially form heterodimers with other NAC genes to function in stress response and plant development (Liang et al., 2019). qRT-PCR analysis found *VcNAC006* and *VcNAC072* to be upregulated in response to drought stress in addition to being correlated, suggesting a potential association and future direction for research (Liang et al., 2019).

There are other potential gene families that are of interest in understanding blueberry responses to drought stress. Recent work by Balboa et al. (2020) compared drought response of three NHB (Bluegold, Brigitta, Elliott) and three SHB cultivars (Biloxi, O’Neal, Sharpblue). Six Late Embryogenesis Abundant (LEA) protein-coding genes and one sequence for a drought-stress marker protein (RD22) were identified and analyzed. The LEA family is another gene family that has been shown in high association with tolerance to dehydration on a cellular level by preventing enzyme inactivation, stabilizing the membrane, binding water molecules, and scavenging free radicals (Balboa et al., 2020). Previous expression profile studies have linked the upregulation of the LEA family to drought-resistant cultivars (Balboa et al., 2020). Analysis of the transcriptome profile of the LEA family showed that dehydrin 1 was upregulated in all cultivars, dehydrin 2 in four cultivars, and dehydrin 3 in all but Biloxi. Additionally, LEA1 was upregulated in all cultivars but Sharpblue, LEA2 in four cultivars, and LEA3 in all cultivars. Differential expression analysis showed that the expression level of RD22 increased in Brigitta and Biloxi but slightly decreased in Sharpblue (Balboa et al., 2020). Different patterns of gene expression (e.g., dehydrin 1-3, LEA1-3, RD22) across cultivars suggested diverse mechanisms existing in blueberries drought responses (Balboa et al., 2020), which suggest the value in screening a wider range of cultivars and a larger collection of genes which could potentially be involved in the molecular network of drought response. Balboa et al. (2020) also stressed the importance of evaluating fruit yield and quality traits to better associate physiological and molecular traits with drought tolerance. Despite several studies assessing the expression levels of targeted gene families, there has been a lack of QTL mapping or genome-wide association studies (GWAS) to further reveal the genetic architecture of blueberry drought tolerance in segregating populations. We will delve further into the methods, challenges, and potential benefits of QTL mapping, GWAS, and molecular breeding for drought tolerance improvement in the discussion.

## 5 Variation in drought tolerance among cultivated blueberries

Adoption of drought tolerant cultivars is the most promising way to cope with increasing drought risks under climate change. As a perennial fruit crop, drought tolerant blueberry cultivars need to endure drought while maintaining plant health, and acceptable fruit quality and yield both in the short-term and long-term. Thus, drought tolerance evaluation of blueberry cultivars must consider fruit-related traits and ideally identify the connections between physiological and biochemical parameters with both short- and long-term plant health and productivity. Nevertheless, few studies compared physiological or biochemical drought responses among commercial cultivars and even fewer evaluated yield or fruit quality traits. Many studies only evaluated physiological or biochemical performance of immature plants without looking into fruit-related traits, which are not directly beneficial for cultivar selection due to the incomplete evaluation of drought tolerance (Balboa et al., 2020). Some studies were limited due to the use of artificial drought conditions such as the use of polyethylene glycol (PEG) in combination with the use of immature plants (Mazurek et al., 2021; Molnar et al., 2022). On account of limited information on comprehensive drought tolerance evaluation, here we will review variation among blueberry cultivars in terms of physiological, biochemical, and growth parameters under drought.

In many crops such as peanut, soybean, common bean, and wheat, cultivars that close their stomata early during drought stress and preserve more water in the soil tend to yield more than cultivars that transpire more and use all soil available water under drought stress (Condon et al., 2004; Vadez et al., 2014; Kaler et al., 2018; Sanz-Saez et al., 2019b; Vadez and Ratnakumar, 2016; Zhang et al., 2022). Cultivars that show lower transpiration usually show high water use efficiency (WUE), which measures the amount of dry matter produced divided by the amount of water consumed (Farooq et al, 2012; Vadez et al., 2014; Vadez and Ratnakumar, 2016). WUE is often used as an indicator of drought tolerance and high WUE can often be reflected by low stomatal conductance or low Δ^13^C in many crops (Condon et al., 2004; Kaler et al., 2018; Sanz-Saez et al., 2019; Zhang et al., 2022). However, selecting for high WUE might reduce yield as WUE has been reported to be negatively correlated with yield in multiple crops such as cotton, wheat, and rice under well-watered conditions (Condon et al., 2004; Blum, 2005; 2009; Polania et al., 2016; Sanz-Saez et al., 2019). In blueberries, Cameron et al. (1989) compared the drought response of two NHB blueberry cultivars, Bluecrop and Jersey, and showed that Jersey had a higher sensitivity to drought than Bluecrop in terms of photosynthesis. However, Cameron et al. (1989) did not observe cultivar variation in stomatal conductance or water use efficiency. Balboa et al. (2020) found genotypic variation in Δ^13^C in an experiment comparing drought responses of container-grown plants of three NHB (Bluegold, Brigitta, Elliott) and three SHB cultivars (Biloxi, O’Neal, Sharpblue). Brigitta showed a lower Δ^13^C while Sharpblue showed a high Δ^13^C compared to other cultivars. An estimated water deficit resistant index (WDRI) was used to identify drought tolerant cultivars, which was calculated as the physiological plant activity of the water deficit treatment (PPA_WD_) divided by the physiological plant activity of the control treatment (PPA_C_). Biloxi (SHB) was suggested to be the most drought tolerant based on a high value of WDRI, however, it remains unclear how these results on immature plants reflect drought tolerance level in mature plants, especially considering the lack of information on yield and fruit quality traits (Balboa et al., 2020).

In many crops, cultivars that show higher fluorescence parameters such as Fv/Fm, ETR, ϕPSII and non-photochemical quenching (NPQ) under drought stress tend to show higher biomass accumulation and yield due to a more drought tolerant photosynthetic system (Li et al., 2009; Boureima et al., 2012; Mishra et al., 2012; Chastain et al., 2014). Estrada et al. (2015) evaluated the performance of three NHB (Bluegold, Elliott, Liberty), three SHB (Bluecrisp, Jewel, Star), and two RE cultivars (Bonita, Powderblue) under drought stress and drought combined with heat stress. Under drought stress alone, cultivars showed differences in chlorophyll fluorescence traits such as Fv/Fm, ETR, ϕPSII and NPQ. In addition, Balboa et al. (2020) showed differences in Fv/Fm among 6 blueberry cultivars: Bluegold, Brigitta, Elliott (NHB) and Biloxi, O’Neal, Sharpblue (SHB). These results suggested the potential to use fluorescence and gas exchange parameters (e.g., stomatal conductance and Δ^13^C) as indicators to select and breed for drought tolerant blueberries. Considering the limited number of genotypes and often immature plants used in previous studies, more research needs to be done in mature blueberry plants of a wide collection of genotypes to correlate physiological parameters with fruit quality traits and yield, which is critical for an accurate evaluation of drought tolerance. Without such validation, cultivar screening only based on physiological parameters will not be sufficient to identify cultivars with good drought tolerance.

Despite the vulnerability of blueberries to drought stress, multiple studies have also shown their ability to quickly recover from drought (Cameron et al., 1989; Améglio et al., 2000; Perrier et al., 2000; Mingeau et al., 2001). Mingeau et al. (2001) observed that when water supply was restored, transpiration returned to control levels within 3-4 weeks for the moderate stressed groups and stem diameter returned to the baseline value after around 10 days. Cameron et al. (1989) studied drought response of NHB cultivars Jersey and Bluecrop and noticed that two weeks after re-watering, stomatal conductance and transpiration of moderate and severe drought groups restored to the level of the control group.

## 6 Discussion: limitations of previous studies and directions for future research

Analysis of previous studies revealed several limitations with blueberry drought tolerance research. First, most studies only focused on shoot traits related to drought without investigating how root anatomy and physiology influences blueberry drought tolerance. Drought studies without a comprehensive evaluation of both shoot and root mechanisms cannot reveal the whole picture of drought tolerance. Second, most studies only evaluated cultivars based on physiological or molecular traits without evaluating fruit yield or quality. As a high-value fruit crop, desirable fruit quality and adequate yield are must-have traits for any cultivar to be commercially adopted and considered drought tolerant. It is therefore essential to correlate physiological traits with fruit traits to identify tolerant genotypes. Third, existing studies only evaluated a small collection of cultivars for drought tolerance, whereas large-scale cultivar screening accompanied by genomic information is needed to reveal diverse drought tolerance mechanisms and underlying quantitative trait loci (QTL) or genes. As a result of limited cultivar evaluation, progress in QTL/gene discovery has been slow for drought tolerance and no molecular-assisted drought tolerance breeding has been reported in cultivated blueberries. Fourth, drought tolerance evaluation requires labor-intensive phenotyping, and a lack of high-throughput phenotyping tools for drought tolerance screening in blueberries is a major bottleneck especially for large-scale cultivar evaluation.

### 6.1 Limited understanding of blueberry root architecture and physiology in relation to drought tolerance

Having a prolific root system is considered a major mechanism for avoiding drought by the uptake of more water from the soil (Erb, 1993; Farooq et al., 2012). Yet, the role of root architecture and physiology in drought tolerance is not well understood in blueberry as most studies focused on the shoot instead of root response to drought. Most, if not all, of the previous studies used container-grown plants in greenhouses or rain-out shelters for the ease of managing drought stress levels. However, a plant’s ability to absorb water in deeper layers of the soil through a large or deep root system cannot be well evaluated when roots are confined in a limited space. For example, rabbiteye blueberries are known for their deeper and tougher root systems and better drought tolerance in field conditions compared to other cultivated ecotypes (Davies and Johnson, 1982; Erb, 1993). However, previous studies focusing mainly on shoot physiological responses were not able to reveal true differences in drought tolerance between RE and SHB blueberries (Estrada et al., 2015; Zhang et al., 2022). Compared to RE cultivars, SHB stems showed stronger mechanical support and safer water transport structures, despite a lower hydraulic conductivity (Zhang et al., 2022). On the other hand, a plant’s ability to uptake water from the soil to alleviate drought stress was omitted in previous studies and therefore the full picture of blueberry drought tolerance is yet to be understood.

Among the few studies investigating the response of blueberry root systems to drought, only root to shoot ratio was studied and contrasting observations were reported. Davies and Johnson (1982) studied drought response in the RE cultivar Bluegem and did not find significant changes in root to shoot ratio under water stress (Davies and Johnson, 1982). Alternatively, Cameron et al. (1989) found that two NHB cultivars Jersey and Bluecrop had a higher root to shoot ratio under severe water stress than well-watered conditions and that Bluecrop partitioned a higher percentage of dry weight to shoots compared to Jersey. The lack of studies evaluating root mechanistic responses to drought is likely due to difficulties in phenotyping roots and the challenges associated with evaluating drought tolerance in field-grown plants as compared to container-grown plants.

In the future, it would be beneficial to investigate the physiological responses of roots to drought and the distribution of roots related to drought tolerance. This could be done, for example, by screening container- or field-grown plants treated with water stress and by studying root distribution and other traits using nondestructive (e.g., X-Ray tomography, mini-rhizotron systems) or destructive approaches (digging roots out using soil core or standard excavation method) (Kuijken et al., 2015; Wasaya et al., 2018). High-throughput phenotyping tools for root image analysis will be needed to improve the efficiency of manual root phenotyping, which can be very labor intensive especially for large datasets (Kuijken et al., 2015; Wasaya et al., 2018; Atkinson et al., 2019). Additionally, integrating soil moisture sensing technologies, such as time domain reflectometry or neutron moisture meters, would facilitate measurements of volumetric water content at various depths over time, providing insight into root physiological responses to dynamic soil moisture conditions (Zhu et al., 2019). High spatial-temporal soil moisture data will help to estimate the water content available to blueberry roots under drought and water uptake by plants during a growing season (Evett et al., 2003; Sprenger et al., 2017).

### 6.2 Insufficient evaluation of drought effects on postharvest traits

Most studies on blueberry drought tolerance focused on shoot physiology without discussing fruit-related preharvest or postharvest traits. For a fruit crop like blueberry, quality and quantity of the final product should be important factors when determining cultivar performance. Evaluation of fruit-related traits for drought tolerance will require more careful experimental planning and larger space compared to studies only focusing on vegetative traits. Only mature plants during the reproductive stage can be used for fruit trait screening which will likely take more space than smaller plants used in some of the vegetative studies. It is also important to consider flowering time variation among cultivars to better plan drought treatments. Artificial chilling may be needed to supplement chilling requirement of some varieties and synchronize flowering time across cultivars to allow the application of drought treatment during the same development stage. Additionally, employment of frost protection using high-tunnels or overhead irrigation is important to protect flower or fruit damage from spring frost events in the field.

### 6.3 Lack of large-scale and comprehensive cultivar screening for drought tolerance

As discussed under section four–variation in drought tolerance among cultivated blueberries, only a few papers reported cultivar evaluation for drought tolerance. Among the reported studies, most focused on evaluating physiological, biochemical, or molecular responses to drought with limited discussion on the identification of drought-tolerant cultivars. For example, Cameron et al., 1989 compared physiological responses of NHB cultivars Jersey and Bluecrop under drought, which showed that Jersey was more sensitive than Bluecrop for photosynthesis but did not differ from Bluecrop for stomatal conductance or water use efficiency (Cameron et al., 1989). A comparison between seedlings of 3 NHB (Bluegold, Brigitta, Elliott) and 3 SHB cultivars (Biloxi, O’Neal, Sharpblue) suggested that Bioloxi was the most drought tolerance based on an estimated water deficit resistant index (WDRI) (Balboa et al, 2020). However, the reliance on immature plants and therefore insufficient assessment of fruit-related traits in Balboa et al. (2020) did not provide adequate evidence to connect physiological parameters with drought tolerance considering fruit quality and yield. In general, there is a lack of large-scale and comprehensive cultivar evaluation for drought tolerance, which hinders the development of drought tolerant blueberry cultivars.

Large-scale drought tolerance evaluation faces many challenges for setting up the controlled drought treatments. First, sufficient greenhouse or rain-out shelter space is needed to allow controlled irrigation, which might not be available for many programs. When rain-out shelters are not available for field trials, no irrigation field trials might be used as an alternative for regions without frequent precipitation during the growing period. However, drought stress levels will be subject to interference of rain, storms, and other weather events. Second, if cultivars with various chilling requirements are screened in the same experiment (e.g., NHB and SHB), cooler space might be necessary to provide sufficient chilling for high-chill cultivars, whereas protection against cold/frost damage might be needed for other cultivars. If cultivars will be evaluated in open field with no protection, it is better to only use cultivars adapted to the local environments to avoid damages from frost, heat, and other stresses. Additionally, large-scale genotype screening will also require phenotyping capacity to evaluate a wide range of shoot, root, and fruit-related traits, which is expensive and labor intensive.

### 6.4 Lack of high-throughput phenotyping tools for blueberry drought tolerance evaluation

To select drought-tolerant, high-yielding blueberry plants more efficiently, modern high-throughput plant phenotyping technologies involving multi-modal imagery, artificial intelligence (AI), sensing, and robotics will be needed. As drought influences the plant’s physiological, morphological, and physio-chemical properties, these changes can be rapidly characterized by using non-destructive imaging and sensing techniques. Ge et al. (2016) used red-green-blue (RGB) and visible near-infrared (VNIR) hyperspectral cameras to phenotype the drought tolerance of two maize genotypes. The results indicated that RGB images accurately predicted the shoot fresh weight, dry weight, and leaf area, whereas the leaf spectra accurately predicted leaf water content. Kim et al. (2020) used RGB, near-infrared (NIR), thermal and fluorescence imagery to quantify drought tolerance in the rice. Results indicated that these imaging modalities were successful in accurately measuring the plant area, water content, plant temperature, and photosynthetic efficiency, respectively. Kim et al. (2015) used short-wave infrared (SWIR) imagery to derive drought-sensitive indices for early detection of water stress. Their result indicated that images in the range of 1400 – 1450 nm are highly correlated with the leaf water content. Beyond-visual range imaging, including hyperspectral, multispectral, fluorescence, and thermal imaging together with artificial intelligence (AI) have also been successfully used as a high-throughput phenotyping modality in other major row crops including maize (Ge et al., 2016; Rehman et al., 2020; Ma et al., 2021; Rehman and Jin, 2022), soybean (Santana et al., 2022), and wheat (Correia et al., 2022). High-throughput phenotyping studies for blueberries have been relatively limited compared to other crops. Recent high-throughput phenotyping research for blueberries has investigated the morphological traits for identifying machine-harvestable genotypes (Patrick and Li, 2017). The study reported a strong correlation between traditional growth indices and quadcopter-based image-derived blueberry volume. The new drought tolerance studies on blueberries are still using the traditional low throughput techniques (Estrada et al., 2015; Balboa et al., 2020; Molnar et al., 2022; Zhang et al., 2022) and therefore need to be updated as has been adopted in other crops.

### 6.5 Limited QTL/genes identified for molecular-assisted breeding

Traditional blueberry breeding relies mainly on phenotypic information for parent/offspring selection, which can take 15–20 years for cultivar release (Hancock et al., 2008). On the other hand, molecular-assisted breeding has a great potential to improve the efficiency of traditional breeding using molecular tools such as marker-assisted selection and genomic selection. Marker-assisted selection refers to the selection of parents or offspring based on a few markers closely linked to the genes or traits of interest (Xu and Crouch, 2008), which is recommended for qualitative traits controlled by a few genes and with a high heritability (Jannink et al., 2010). Genomic selection, which aims to predict the performance of an individual based on simultaneously estimated effects of all markers across the genome, is recommended for quantitative traits controlled by many and small-effect genes (Jannink et al., 2010). Both marker-assisted selection and genomic selection offers the opportunity to identify optimal individuals using marker information, potentially speeding up drought tolerance breeding by substituting challenging and costly phenotyping protocols.

The potential of molecular breeding to enhance drought tolerance is impeded by a lack of understanding on the genetic basis of drought tolerance in blueberries. The implementation of marker-assisted selection requires preidentified QTL or genes closely related to the trait of interest, often through QTL mapping or GWAS (Edger et al., 2022). In blueberries, QTL have been identified for traits related to fruit quality, machine harvesting, and climatic adaptation (Edger et al., 2022). Nevertheless, no QTL mapping or GWAS have been reported for drought tolerance, largely due to similar challenges encountered in large-scale cultivar evaluation for drought tolerance. The blueberry community does have rich genomic resources and tools for QTL/gene discovery, including high-quality reference genomes, next-generation genotyping platforms, linkage maps, and analysis software for polyploid species (Edger et al., 2022). If challenges related to large-scale drought tolerance evaluation can be addressed, QTL mapping or GWAS can be made possible by conducting drought evaluation on segregating mapping populations or diverse panels. As a result, a deeper understanding of the genetic architecture of drought tolerance will help researchers to identify optimal breeding strategies: marker-assisted selection if large-effect QTL or genes are discovered, or genomic selection if drought tolerance is highly quantitative in nature.

In addition to QTL mapping and GWAS, transcriptomics is another useful tool to advance gene discovery for drought tolerance in blueberries, especially when used alongside other QTL/gene mapping methods. Transcriptomics involves the study of transcriptome, which refers to the complete set of RNA transcripts or messenger RNA (mRNA) that are produced by the genome under specific conditions or in a specific cell (Vulimiri et al., 2014). Advances in next-generation RNA sequencing technologies have provided researchers with powerful tools to study gene expression patterns in greater detail, with higher accuracy, and at a more affordable price (Stark et al., 2019). As discussed in section 3, which focuses on blueberries’ molecular response to drought, gene expression analysis of targeted transcription factor and gene families has revealed their significant role in blueberries’ drought responses. For example, MYB TF family (Zhang et al., 2020), NAC TF family (Liang et al., 2019), and LEA family (Balboa et al., 2020) have all been implicated. Since drought tolerance involves complicated metabolic and signaling pathways, future studies should analyze transcriptome rather than single gene families to better reveal the molecular mechanism of drought tolerance. Additionally, transcriptome analysis has been combined with QTL mapping to facilitate candidate gene discovery such as for leaf development in alfalfa (Jiang et al., 2022), hypocotyl elongation in rapeseed (Luo et al., 2017), pericarp thickness in sweet corn (Wu et al., 2020), and heat-tolerance in tomato (Wen et al., 2019) to facilitate candidate gene discovery. Joint QTL mapping and transcriptome analysis can be potentially useful for dissecting the genetic architecture of drought tolerance in blueberries. While molecular information can be instrumental for cultivar development, it is essential to develop a systematic approach to improve drought tolerance while maintaining an ideal level of other traits such as yield, fruit quality, and disease tolerance.

### 6.6 Future directions for improving blueberries for drought tolerance

Drought-tolerant blueberry cultivars must satisfy multiple criteria. At the minimum, they should demonstrate the resilience to drought stress and the capacity for rapid recovery from any damage incurred. Furthermore, it is essential for blueberry plants to maintain overall health, ensuring sustainable production over the long term. Exceptional cultivars go beyond these basic requirements, exhibiting the ability to uphold acceptable fruit quality and yield even under drought stress, all while preserving their long-term health and productivity.

To breed blueberries with ideal drought tolerance, comprehensive research efforts are essential across several key areas to effectively address challenges in blueberry drought research: (1) drought tolerance evaluation to include a broader range of traits, such as root architecture and fruit-related performance under drought stress, to establish stronger association between physiological and molecular signals with drought tolerance mechanisms; (2) large-scale drought tolerance screening across diverse blueberry germplasm to uncover various drought tolerance mechanisms and valuable genetic resources; (3) high-throughput phenotyping for drought-related traits to enhance the efficiency and affordability of drought phenotyping; (4) identification of genetic architecture of drought tolerance using various mapping technologies and transcriptome analysis; (5) tools to facilitate molecular-assisted breeding for drought tolerance, such as through marker-assisted selection and genomic selection; and (6)investigation of the interactions between drought and other stresses, such as heat and mineral soil conditions, to develop stress resilient genotypes.

## Author contributions

SR: Conceptualization, Formal analysis, Investigation, Visualization, Writing – original draft, Writing – review & editing. AS-S: Writing – original draft, Writing – review & editing. CL: Writing – review & editing. TR: Writing – original draft, Writing – review & editing. SB: Writing – original draft, Writing – review & editing.
