# Supplementary material for: Review on blueberry drought tolerance from the perspective of cultivar improvement
Source: Front Plant Sci. 2024 May 14;15:1352768. doi: 10.3389/fpls.2024.1352768 (PMC11130474; doi:10.3389/fpls.2024.1352768)
Supplement: Supplementary file 1 [file DataSheet_1.zip › Table S1-S3.DOCX]

**Supplementary materials**

**Table S1. Summary of drought studies related to the physiological and biochemical responses of blueberries under drought**

| **Reference** | **Materials and methods** |
| --- | --- |
| **Davies and Johnson, 1982**  Water stress, growth, and critical water potentials of rabbiteye blueberry | **Cultivar:**  Bluegem (RE)  **Plants:**  Rooted cuttings, unknown age, 3785 ml (1 gallon) pot.  **Treatment:**  Control: watered every 2 days  Mild: watered every 7 days, baxial diffusion conductances (g_s_) dropped to 80%  Severe: watered every 10 days, g_s_ dropped to 50%  **Measured traits:**  Dry weight of root, stem and leaves, root to shoot ratio, leaf water potential,  relative water content, transpiration ratio  **Major findings:**   1. Root: shoot ratio was not altered significantly by water stress, which indicates that growth of roots and shoots was balanced during water stress. 2. Critical water potential for stomatal closure was estimated to be -2.2 MPa. 3. Relative water content decreased an average of 6.4% for every 1 MPa change in water potential. 4. Transpiration ratio: 222g of water transpired per g dry matter produced. |
| **Cameron et al., 1989**  The influence of soil moisture stress on the growth and gas exchange characteristics of young highbush blueberry plants (*Vaccinium Corymbosum* L.) | **Cultivar:**  Jersey, Bluecrop (NHB)  **Plants:**  Two-year-old containerized plants maintained in a greenhouse.  **Treatment:**  Control: well-watered, moisture ≥ –30 cbars  Moderate stress: moisture between -60 and -70 cbars  Severe stress: moisture ≤ -80 cbars  **Measured traits:**  Photosynthesis rate, transpiration, stomatal conductance, whole plant and individual leaf areas, total number of leaves, dry weight per leaf unit area, leaf shape, dry weight of shoots and roots  **Major findings:**   1. Gas exchange rates were significantly reduced under drought but recovered after plants were rewatered 2. Leaf numbers and leaf are decreased under drought. 3. More biomasses were allocated to the root systems of drought-stressed plants. |
| **Ameglio et al., 2000**  Water relations of highbush blueberry under drought conditions | **Cultivar:**  Bluecrop (NHB)  **Plants:**  Nine-year-old, container-grown in greenhouse  **Treatment:**  Drought: 10 days without water  **Measured traits:**  Minimum and predawn leaf water potential, stomatal conductance, daily transpiration  **Major findings:**   1. Leaf water potential decreased in drought-treated plants. Minimum leaf water potential stayed at -0.8 MPa towards the end of drought while predawn leaf water potential continued to drop, which indicates that Bluecrop is an isohydric cultivar. 2. Maintaining the Ψ_Min_ around the cavitation threshold made it possible to protect the plant against strong embolism. Stomatal regulation is therefore very important for this plant since the threshold of cavitation is reached rapidly. 3. Stomatal conductance and transpiration rate rapidly decreased during drought and quickly recovered after rehydration. 4. Embolism increases very rapidly below -1.2 MPa |
| **Perrier et al., 2000**  Effects of water stress on transpiration, radial growth and yield in highbush blueberry | **Cultivar:**  Bluecrop (NHB)  **Plants:**  Seven-year-old plants growing in 25 L containers.  **Treatment:**  Moderate stress: transpiration index = 0.65 of control  Severe stress: transpiration index = 0.35 of control  Treatment was applied for two weeks during various growth stages: fruit growth, maturation, harvest, post-harvest  **Measured traits:**  Transpiration, stem diameter, yield, average fruit weight  **Major findings:**   1. Drought stress during fruit growth stage has the highest impact on production in the same year. 2. Post-harvest drought stress had the largest impact on production in the next year, mainly by reducing the number of berries. 3. Transpiration and stem diameters decreased during drought, both of which recovered to various degrees after drought treatment ended. |
| **Mingeau et al., 2001**  Evidence of drought-sensitive periods from flowering to maturity on highbush blueberry | **Cultivar:**  Bluecrop (NHB)  **Plants:**  Five-year-old plants grown in 25 L containers. An automated rain protection system was used to control water supply.  **Treatment:**  Moderate stress: transpiration index = 0.65 of control  Severe stress: transpiration index = 0.35 of control.  **Measured traits:**  Transpiration, shoot elongation, stem diameter, yield, bud count (second year), number of fruits, average fruit weight  **Major findings:**   1. Water stress during fruit growth and maturation reduced yield by around 40% and reduced average fruit weight by close to 17% in moderate stress and up to 39% in severe stress in the same season. 2. Post-harvest drought had the largest impact on the number of berries (23- 43% fewer berries), average berry weight (21-59% higher), and yield (27% less in severe drought) in the next season. |
| **Rho et al., 2012**  Limitation factors for photosynthesis in ‘Bluecrop’ highbush blueberry (*Vaccinium corymbosum*) leaves in response to moderate water stress | **Cultivar:**  Bluecrop (NHB)  **Plants:**  Three-year-old plants growing in 11 L (3 gallon) container-grown  **Treatment:**  Control: 1 L of water/day  Drought: non-irrigated.  Treatments started when shoots expanded approximately ten leaves, lasted for 3 months  **Measured traits:**  Gas exchange  Chlorophyll fluorescence  **Major findings:**   1. The decrease in CO_2_ assimilation following water stress may be caused by a decrease in mesophyll conductance rather than a decrease in stomatal conductance. 2. Mesophyll limitation than stomatal and biochemical limitations mainly downregulated the photosynthesis in the leaves of ‘Bluecrop’ blueberry shrubs during moderate water stress. |
| **Chen et al., 2017**  Spermidine induces physiological and biochemical changes in southern highbush blueberry under drought stress | **Cultivar:**  Unknown  **Plants:**  1-year-old seedlings growing in pots 25 cm in diameter and 20 cm in height.  **Treatment:**  Control: 80-85% field capacity  Moderate drought: 45-50% field capacity  Severe drought stress: 20-25% field capacity. Treatment lasted for 16 days.  **Measured traits:**  Photosynthetic rate (Pn), relative water content (RWC), total chlorophyll content (Chl), specific leaf weight (SLW), malondialdehyde (MDA) content, relative electrolyte conductivity (REC), antioxidant enzymes activities  **Major findings:**   1. Relative water content, total chlorophyll content, net photosynthetic rate, specific leaf weight, 3-acetic acid (IAA) content, gibberellic acid (GA) content, polyamine (PA) content decreased as drought prolonged and as drought severity increased. 2. MDA content, REC, superoxide dismutase (SOD) activities, peroxidase (POD) activities, and total soluble sugar contents of blueberry leaves, abscisic acid (ABA) content increased as drought level increased and drought treatment prolonged. 3. Spermidine plays a role in alleviating the negative effects of drought stress on plants. |
| **Panta et al., 2001**  Effect of cold and drought stress on blueberry dehydrin accumulation | **Cultivars:**  Bluecrop (NHB), Premier (RE)  **Plants:**  Four-year-old container-grown plants maintained in a greenhouse  **Treatment:**  Control: watered daily  Drought: no water for 34 days  **Major findings:**   1. Dehydrins accumulated with both cold and drought stress. 2. Relative amount of dehydrins accumulated in stems, roots, and leaves varied between cold and drought-stressed plants. 3. In drought-stressed plants, dehydrins accumulates before significant changes in RWC and their levels did not closely correlate with RWC. |
| **Mazurek et al., 2021**  Differences in response to drought stress among highbush blueberry plants  propagated conventionally and by tissue culture | **Cultivar:**  Brigitta Blue (NHB)  **Plants:**  Softwood propagated plants and tissue cultured plants grown in 100 ml pots.  **Treatment:**  Induced drought using polyethylene glycol (PEG) at 3% and 7%  Duration of treatment: 21 days  **Measured traits:**  Shoot elongation, leaf weight, leaf water content, chlorophyll fluorescence parameters  **Major findings:**   1. Drought stress negatively affected shoot elongation, chlorophyll fluorescence parameters, leaf water content, leaf weight, and chlorophyll content. 2. Propagation methods have effect on plants’ physiological response to drought.   **Limitations:** Very small plants. Drought stress was induced using polyethylene glycol. It is unclear how well these results corelate with drought stress in mature plants under real drought conditions. |

**Table S2. Summary of drought studies related to molecular mechanisms**

| **Reference** | **Materials and methods** |
| --- | --- |
| **Liang et al., 2019**  Identification and Expression of NAC Transcription  Factors of *Vaccinium corymbosum* L. in Response to  Drought Stress | **Cultivars:**  Bluecrop (NHB)  **Plants:**  Seedlings about 20cm in height.  Greenhouse.  **Treatment:**  Control: well-watered  Moderate drought: no water for 20 days  Severe drought: no water for 40 days  **Major findings:**   1. Chlorophyll fluorescence parameters of leaves were significantly inhibited under drought. 2. Abscisic acid (ABA) content increased, gibberellic acid (GA_3_) and indole acetic acid (IAA) content decreased. 3. Sixty two NACs were differently expressed in leaf and root under drought, 14 NACs among which were significantly correlated with the expression of other NAC genes. |
| **Wang et al., 2021**  Genome-wide analysis of MYB transcription  factors of *Vaccinium corymbosum* and their  positive responses to drought stress | **Cultivars:**  Bluecrop (NHB)  **Plants:**  Tissue-culture seedlings about 18cm in height  Greenhouse  **Treatment:**  Water was withheld until soil water content (SWC) reached pre-determined levels  Control: SWC 75-80%  Moderate drought: SWC 55-60%  Severe drought: SWC 30-35%  **Major findings:**   1. A total of 102 MYB differentially expressed genes (DEGs) were identified in drought-stressed compared to control plants with roughly half expressed in shoots and half in roots. 2. Ten key *VcMYB* genes were predicted to be involved in reactive oxygen species (ROS) pathway and seven in leaf regulation under drought stress. |
| **Wang et al., 2022**  Comparative transcriptome profiling reveals the defense pathways and mechanisms in the leaves and roots of blueberry to drought stress | **Cultivars:**  Bluecrop (NHB)  **Plants:**  3-month-old uniform seedlings about 18cm in height  Greenhouse  **Treatment:**  Water was withheld until soil water content (SWC) reached pre-determined levels  Control: SWC 75-80%  Moderate drought: SWC 55-60%  Severe drought: SWC 30-35%  **Major findings:**   1. Gene expression patterns differed between leaves and roots of blueberries under drought stress 2. Most DEGs were associated with signal transduction, transcriptional regulation, and metabolism. 3. The plant hormone signal transduction pathway was the pathway with most core genes in leaves whereas the MAPK signaling pathway was more important for roots. 4. POD activity, ABA content, and anthocyanin biosynthesis were significantly increased in leaves and roots under drought. |
| **Zhang et al., 2020**  *VcMYB4a*, an R2R3-MYB transcription factor from Vaccinium corymbosum,  negatively regulates salt, drought, and temperature stress | **Cultivars:**  Bluecrop (NHB), Northland (NHB)  **Plants:**   1. Nine-year-old plants grown in orchard (Bluecrop) 2. One-year-old plants from tissue culture in growth chamber (Bluecrop) 3. White loose calli (Northland)   **Treatment:**   1. Control 2. 20% Polyethylene glycol 6000 for 0h, 0.5h, 1h, 2h, 4h, and 6h 3. Calli cultured in MWPM medium with PEG-6000 for one day at 0%, 10%, 20%, 30%, and 40%   **Major findings:**   1. VcMYB4a expression was downregulated by salt, drought, and cold treatment, but was induced by freezing and heat. 2. Overexpression of VcMYB4a in blueberry callus enhanced sensitivity to salt, drought, cold, freezing, and heat stress. |

**Table S3. Summary of drought studies related to variation of drought tolerance across genotypes**

| **Reference** | **Materials and methods** |
| --- | --- |
| **Estrada et al., 2015**  Fluorescence phenotyping in blueberry breeding for genotype selection under drought conditions, with or without heat stress | **Cultivars:**  Bluegold, Elliott, Liberty (NHB)  Bluecrisp, Jewel, Star (SHB**)**  Bonita, Powderblue (RE)  **Plants:**  Plants are 2-3 years old, grown in 20 L (5 gallon) containers in a greenhouse.  **Treatment:**  Control: continuous irrigation  Drought: one third of the control water volume  Heat stress was also investigated.  **Measured traits:**  Chlorophyll fluorescence, stem water potential, chlorophyll content, leaf temperature, SPAD  **Major findings:**   1. Under drought stress alone, cultivars showed differences in most chlorophyll fluorescence traits, but only the quantum yield of energy conversion of non-photochemical quenching [Y(NPQ)], stem water potential (ψ_S_), and interactions between cultivars and irrigation treatments were significant. 2. Cultivars differ in the efficiency of photosystem II (PSII) 3. Cultivars showed different responses in the efficiency and operation of PSII when treated with both drought and heat stresses. |
| **Balboa et al., 2020**  Integration of physiological and molecular traits would help to improve the insights of drought resistance in highbush blueberry cultivars | **Cultivars:**  Elliot, Bluegold, Brigitta, Sharpblue (NHB)  Biloxi, O’Neal (SHB)  **Plants:**  Seedlings from tissue culture, unknown plant age, very small plants grown in 800ml (0.2gallon) pots maintained in a greenhouse.  **Treatment:**  Control: 300ml water every other day  Drought: 150ml water every other day  **Measured traits:**  RWC, chlorophyll fluorescence (Fv/Fm), proline content, carbon isotopic discrimination (Δ^13^C), gene expression for LEA proteins  **Major findings:**   1. Fv/Fm, RWC, and Δ^13^C decreased under drought stress while proline content increased. 2. Differences in the estimated water deficit resistance index were observed among six cultivars.   **Limitations:** Plants are very small. Drought tolerance level of seedlings might not reflect that with mature plants. |
| **Molnar et al., 2022**  Response of the five highbush blueberry cultivars to in vitro  induced drought stress by polyethylene glycol | **Cultivars:**  Bluecrop, Brigitta Blue, Duke, Goldtraube, Hortblue Petite (NHB)  **Plants:**  Tissue culture plants growing in a culture medium. Plants are very small, but the actual sizes were not described.  **Treatment:**  PEG 6000 concentrations varied from 0 (g/L) to 50 (g/L) with an increment of 10 (g/L).  Treatment duration: 12 weeks.  **Major findings:**   1. Drought stress had a negative impact on shoot length, chlorophyll content and carotenoid content for all five cultivars. 2. The cultivar Goldtraube was suggested to be most drought tolerant, but its not clear how this conclusion was made and how much it reflects drought tolerance in mature plants and in natural drought conditions   **Limitations:**  Very small seedling plants. Drought stress was induced using polyethylene glycol. It is unclear how well these results corelate with drought stress in mature plants under real drought conditions. |
| **Zhang et al., 2022**  A more drought resistant stem xylem of southern highbush than rabbiteye blueberry is linked to its anatomy | **Cultivars:**  O’Neal, Misty (SHB), Brightwell, Tifblue, Climax (RE)  **Plants:**  One-year-old, post-fruit-stage branches were studied.  **Measured traits:**  Xylem water potential at 12, 50, and 88% loss of hydraulic conductivity, branch and sapwood hydraulic conductivity, vessel diameter, vessel density, etc.  **Major findings:**   1. SHB has less conductive but safe xylem than RE. 2. SHB has smaller vessels but higher conduit wall reinforcement, wood density, and vessel-grouping index than RE. |
